# Quantifying gait compensation in knee osteoarthritis using smartphone-based motion capture (OpenCap)

**DOI:** 10.3389/fspor.2025.1674133

**Published:** 2025-11-13

**Authors:** Jia-Jing Xu, Yi Xu, Peng-Fei Shi, An Shao, Jin-Tao Pan, Xi-Ping Ren, Shi-Jia Wang, Yong-Wei Zhou, Lian-Ping Jiang, Qi-Ning Yang

**Affiliations:** 1 Jinhua Municipal Central Hospital, Zhejiang University, Jinhua City, Zhejiang, China; 2 Department of Natural Resources, Faculty of Geo-Information Science and Earth Observation, University of Twente, Enschede, Netherlands; 3 College of Physical Education and Health Sciences, Zhejiang Normal University, Jinhua, China

**Keywords:** biomechanical gait analysis, knee osteoarthritis, OpenCap, smartphone-based motion capture system, range of motion

## Abstract

**Background:**

Knee osteoarthritis (KOA) is one of the most prevalent degenerative joint diseases, and its pathological features often lead to abnormal gait patterns and limited joint mobility. These changes induce various degrees of lower-limb compensatory mechanisms that significantly affect patients’ quality of life and increase their risk of falls. An accurate and objective assessment of these gait changes and compensatory strategies is critical for clinical diagnosis, monitoring of disease progression, and the formulation of rehabilitation strategies.

**Objective:**

This study aims to investigate the compensatory mechanisms of lower limb kinematics in KOA patients during walking using a markerless motion capture system—OpenCap—and toevaluate its feasibility and accuracy in a clinical environment.

**Methods:**

A total of 33 KOA patients and 78 healthy control participants were enrolled. Two smartphones were used to record videos of participants walking along a flat path, and OpenCap was employed to calculate spatiotemporal gait parameters and 3D joint kinematics. Data were statistically analyzed to determine differences in gait features between KOA patients and controls.

**Results:**

KOA patients had significantly reduced walking speed and stride length, and exhibited increased step width, reduced knee flexion-extension range, and greater pelvic tilt and hip internal rotation during certain phases of the gait cycle. These findings reflect biomechanical compensation strategies related to joint pain, instability, or restricted mobility. OpenCap provided reliable and accurate motion data and demonstrated strong potential for hospital-based gait assessments due to its low cost and ease of setup.

**Significance:**

This study demonstrated that OpenCap effectively captures KOA-related gait abnormalities and compensatory joint movements. Its low cost and ease of use support its application in hospital settings for dynamic evaluation and rehabilitation planning.

## 1 Background

Knee osteoarthritis (KOA) is a progressive degenerative joint disorder that primarily affects the articular cartilage, subchondral bone, and surrounding soft tissues (1). The global burden of KOA is rising, largely due to population aging, increasing rates of obesity, and sedentary lifestyles (2). Epidemiological evidence shows that KOA has one of the highest incidences and disability rates among musculoskeletal diseases, particularly in populations over the age of 60 (3). Beyond its impact on individual health, KOA contributes to chronic pain, functional limitations, and reduced mobility (4), which lead to lost productivity and increased consumption of medical resources, thereby exacerbating socioeconomic burdens.

The Kellgren–Lawrence (KL) classification is widely used in the standardized assessment and management of KOA, it enables grading of disease severity based on x-rays and guides surgical treatment (5); however, its limitation is that the x-ray-based grading may not be fully consistent with the patient's subjective levels of pain and functional impairment. Self-reported scales (such as WOMAC, KOOS, and VAS) are common tools for assessing KOA, they can directly reflect patients' subjective feelings (6, 7). Given the limitations of traditional assessment methods, there is an urgent need for relatively objective and more reliable kinematic analysis to evaluate KOA. KOA is clinically characterized by pain, stiffness, swelling, reduced range of motion, and decreased muscular control, all of which can result in compensatory gait patterns (8). These patterns may manifest as reduced gait speed, shortened stride length, altered joint angles, or asymmetric loading between limbs (9, 10). A meta-analysis has reported that gait speed in KOA patients is on average reduced by 15%–20% compared to healthy individuals (11). Quantitative analysis of these gait characteristics is essential for early diagnosis, monitoring disease progression, evaluating postoperative outcomes, and developing targeted rehabilitation programs. However, traditional gait analysis systems, such as marker-based optical motion capture (e.g., Vicon) and inertial measurement unit (IMU) systems, offer precise kinematic measurements but face barriers to widespread clinical use due to high costs, complex operation, space requirements, and time-consuming marker placement and calibration procedures (9, 12). These challenges limit their feasibility for routine outpatient assessment or high-throughput screening.

Recent advances in computer vision and machine learning have enabled the development of markerless motion capture systems that estimate human motion using only video input. Among these, OpenCap has attracted significant attention for its open-source design, low equipment cost, and ease of use (13). OpenCap requires only two standard smartphones, using deep learning–based models (such as OpenPose and HRNet) for 2D joint detection, followed by 3D pose reconstruction and musculoskeletal modeling via OpenSim (14). Its smartphone deployment cost is estimated at just 1/200 that of advanced systems like Theia3D (15), making it highly accessible for clinical and research use.

However, most existing validations have focused on technical performance under controlled laboratory conditions. Research on the application of OpenCap in real hospital environments, especially its ability to detect disease-specific kinematic changes and compensatory strategies in KOA patients, remains limited. Although OpenCap's low cost and ease of use make large-scale gait analysis studies feasible, its reliability in complex clinical scenarios still requires further verification. Therefore, the aim of this study is to explore lower-limb compensatory kinematics in patients with KOA using the OpenCap system and to evaluate its feasibility, accuracy, and clinical utility. By comparing the spatiotemporal parameters and joint motion features of KOA patients and healthy individuals, we aim to assess the ability of OpenCap to reveal biomechanical adaptations associated with knee osteoarthritis. Additionally, we seek to explore its application in hospital settings for assessing disease severity, analyzing postoperative recovery, and guiding rehabilitation programs.

## 2 Materials and methods

### 2.1 Participants

This prospective cohort study enrolled 33 patients diagnosed with medial compartment knee osteoarthritis (KOA) and 78 healthy control participants matched for age and sex. KOA patients were required to meet clinical diagnostic criteria, confirmed radiographically with K&L grades ≥ 2. Inclusion criteria for the KOA group comprised: (1) ability to ambulate independently without assistive devices; (2) presence of knee pain or stiffness on most days in the past month; and (3) absence of recent lower limb surgeries or pathologies. The exclusion criteria, applied uniformly to both groups, included a history of lower extremity musculoskeletal surgery or traumatic injury, diagnosed neurological disorders (e.g., Parkinson's disease, stroke), impaired independent ambulation or high fall risk, and unwillingness to participate. The healthy control group included individuals with no history of lower limb joint pain, surgery, or diagnosed joint disease. All participants provided written informed consent prior to study participation. The protocol was approved by the Institutional Ethics Committee (Approval No. 20242170101).

### 2.2 Experimental setup

Gait assessments were conducted adhering to the OpenCap software and hardware configuration protocol developed at Stanford University. Prior studies have validated OpenCap's accuracy in healthy individuals, estimating joint angles with mean absolute errors (MAE) of less than 5° for a range of activities including walking, squatting, rising from a chair, and performing drop jumps (13, 16). Wang et al. evaluated OpenCap in patients with KOA and reported acceptable consistency with marker-based systems, the results showed that OpenCap exhibited good consistency with the optical system in measuring core parameters (e.g., peak knee flexion-extension angle, knee angle range during gait), with intraclass correlation coefficient (ICC) values mostly between 0.80 and 0.95 (17). The Bland-Altman plot indicated a small mean bias (<3°) between the two sets of measurements, and the 95% limits of agreement (LOA) fell within the clinically acceptable range. Repeated measurements on the same patients revealed that OpenCap had a coefficient of variation (CV) <5%, with reliability comparable to the optical system, demonstrating good stability in repeated measurements.

Video recording was conducted in the same closed and spacious room within the hospital to avoid errors caused by environmental interference. Data were collected using two iPhone 12 devices equipped with rear cameras recording at 60 frames per s (fps) and a resolution of 720 × 1,280 pixels. The cameras were positioned approximately 2.5 meters apart at angles between 30° and 45° relative to the participant's walking direction, at heights ranging from 1.0 to 1.2 meters to align with the pelvis. Before data collection, a calibration procedure was performed using a printed A4-sized (210 mm × 297 mm) chessboard pattern to ensure accurate spatial alignment. Lighting conditions were standardized to reduce visual artifacts and improve pose detection fidelity.

**Figure 1:**
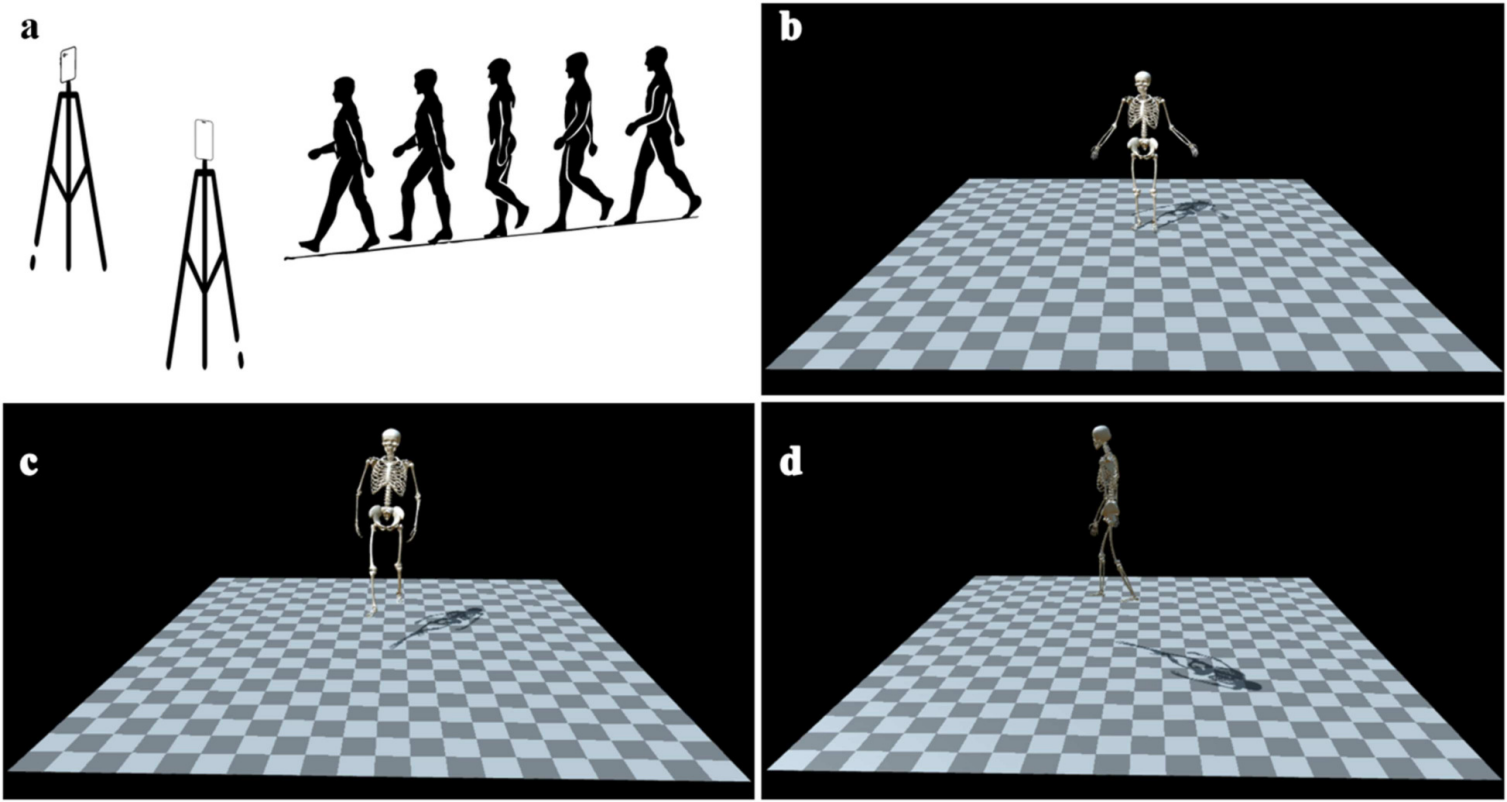
A schematic diagram of video-based human motion dynamics analysis (OpenCap). **(a)** Two smart phones are placed in an indoor hospital environment, and each participant faced the video recording device to complete the collection of standing posture and the video recording of walking trails. **(b)** 3D kinematic model of the standing posture. **(c)** Front view of the walking 3D kinematic model. **(d)** Lateral view of the walking 3D kinematic model.

As shown in Figure 1, participants first assumed a static barefoot neutral standing posture, recorded for musculoskeletal scaling in OpenSim via OpenCap's Scale tool. This step enabled the creation of individualized biomechanical models by matching anatomical landmarks to the participant's anthropometric dimensions. Participants then walked barefoot along an approximately 6-meter level walkway at a self-selected comfortable speed. Each participant completed three valid walking trials while facing the cameras. Walking toward the cameras was chosen based on prior validation indicating reduced joint angle estimation errors in this configuration (RMSE = 6.0° vs. 8.1°) (17). To standardize foot-ground interactions, all participants performed the trials barefoot, mitigating variability introduced by footwear. Previous research has shown that footwear can significantly affect gait parameters such as stride length variability, stride time variability, and step width, due to differences in sole thickness and stiffness (18). Participant safety was ensured throughout all walking trials.

### 2.3 Data acquisition and processing

Video recordings were uploaded to the OpenCap platform for automated processing. Anatomical keypoints were detected in two-dimensional frames using both OpenPose and HRNet algorithms, representing state-of-the-art deep learning–based human pose estimation methods. Corresponding keypoints from both camera views were synchronized and triangulated to reconstruct three-dimensional joint trajectories (19). Dynamic kinematics were computed via OpenSim's inverse kinematics framework, which estimated joint rotations by fitting a musculoskeletal model to the reconstructed 3D marker trajectories. Standing posture recordings were used to scale the musculoskeletal model for each participant, aligning bone segment lengths to individual anthropometry for improved modeling accuracy.

Trajectory data were smoothed using a Savitzky–Golay filter (window length = 30 frames; polynomial order = 3) to reduce high-frequency noise while preserving physiologically meaningful motion patterns. Heel-strike and toe-off events were automatically detected from vertical displacement profiles of heel and toe markers. A complete gait cycle was defined from the initial heel-strike of one leg to the subsequent ipsilateral heel-strike. Each gait cycle was time-normalized to 100% using linear interpolation to facilitate phase-dependent analyses. A quality assurance process involved systematically excluding gait cycles with abnormal timing, marker discontinuities, or biomechanically implausible trajectories. Common reasons for rejection included camera occlusion, participant movement outside the capture zone, or pose detection failures due to lighting variability. Following filtering, 362 gait cycles from the KOA group and 882 from the control group were retained for analysis.

### 2.4 Joint kinematic and spatiotemporal parameter extraction

Joint kinematic parameters were estimated using OpenCap's OpenSim-based inverse kinematics solver, yielding three-dimensional rotational trajectories for the pelvis, hip, knee, and ankle joints across the gait cycle. Degrees of freedom analyzed included sagittal (flexion/extension), frontal (abduction/adduction), and transverse (internal/external rotation) planes. Specific parameters extracted included pelvic tilt, pelvic list, pelvic rotation; hip flexion/extension, adduction/abduction, internal/external rotation; knee flexion/extension; ankle dorsiflexion/plantarflexion; and subtalar inversion/eversion. All kinematic curves were smoothed using the Savitzky–Golay filter to minimize noise while retaining physiologically relevant motion patterns.

Spatiotemporal parameters were derived by tracking heel and toe markers, including gait speed (m/s), stride length (m), step width (cm), cadence (steps/min), double support (% of gait cycle), and step length asymmetry (%). Double support was defined as the proportion of the gait cycle with both feet in ground contact. Step length asymmetry was computed as the percentage difference between left and right step lengths relative to their average. All kinematic and spatiotemporal variables were normalized to 100% of the gait cycle for consistent inter-subject comparison. Outlier detection employed a ±3 standard deviation threshold across the pooled dataset, with visual inspection to exclude values arising from tracking artifacts rather than true biomechanical variation. Retained data were averaged across valid gait cycles per participant to yield representative values for statistical analyses.

### 2.5 Statistical analysis

Baseline characteristics of the KOA and control groups were compared to ensure equivalence and reduce confounding. Data normality was assessed via the Shapiro–Wilk test, and variance homogeneity using the Levene test. Continuous variables were analyzed using independent *t*-tests assuming unequal variances, while categorical variables were assessed using chi-squared tests. Significance thresholds were reported at *p* < 0.05, *p* < 0.01, and *p* < 0.001 to denote the strength of observed differences. To enhance data reliability, a systematic filtering strategy was employed rather than selecting only visually optimal gait cycles. Cycles exhibiting excessive error due to sensor noise, signal loss, or biomechanically implausible movement were excluded to minimize the impact of outliers without introducing selection bias. The final dataset comprised 882 gait cycles from the control group and 362 from the KOA group. All statistical analyses were performed using Python 3.9.16, employing libraries such as NumPy and Pandas for data handling, SciPy for inferential statistics, and Matplotlib for data visualization. This analytical framework ensured robust, reproducible, and interpretable comparisons between the experimental and control groups.

## 3 Results

### 3.1 Patient characteristics

A total of 111 participants were enrolled in this study, including 33 patients diagnosed with medial compartment KOA (11 males and 22 females; mean age: 68.82 ± 5.54 years) and 78 healthy control subjects (30 males and 48 females; mean age: 66.25 ± 6.78 years). Reliability and validity testing was conducted in the study, with preset parameters including an effect size of Cohen's *d* = 0.5 and a significance level of *α* = 0.05, the statistical power (1 −* β*) was calculated to be approximately 85%. There were no statistically significant differences between the groups in terms of age, sex distribution, or body mass index (BMI) (*p* > 0.05; see Table 1). Among KOA patients, 14 presented with left-sided involvement and 19 with right-sided disease. Kellgren–Lawrence (KL) grading revealed 5 patients at grade 2, 16 at grade 3, and 12 at grade 4. Varus deformity was present in 29 patients (mean varus angle: 12.75 ± 3.54°), while 4 patients exhibited valgus deformity (mean valgus angle: 9.01 ± 4.29°). Excluding equipment setup and calibration time, each participant completed the standing posture calibration and three walking trials in approximately 6–8 min.

**Table 1:** The demographic characteristics and basic gait data of the control and experimental groups.

Characteristic	Control (*n* = 78) Mean (SD)	Experiment (*n* = 33) Mean (SD)	*p*-Value
Age (years)	68.82 (5.54)	66.25 (6.78)	0.253
Sex (male/female)	30/48	11/22	0.671
BMI (kg/m^2^)	23.94 (2.77)	24.84 (3.76)	0.167
Gait speed (m/s)	1.24 (0.37)	0.87 (0.44)	0.004*
Stride length (m)	1.32 (0.55)	0.97 (0.62)	0.084
Step width (cm)	8.87 (2.10)	13.23 (3.92)	<0.001*
Cadence (step/min)	112.50 (12.33)	98.30 (18.89)	0.019*
Double support (%cycle)	22.68 (3.27)	38.54 (10.26)	<0.001*
Step length asymmetry (%)	2.75 (1.17)	7.67 (4.83)	<0.001*

* *p*-value < 0.05 is considered statistically significant.

### 3.2 Kinematic characteristics of experimental and control groups

#### 3.2.1 Basic gait parameters

Table 1 summarizes the basic gait parameters. The KOA group demonstrated significantly reduced gait speed (0.87 ± 0.44 m/s vs. 1.24 ± 0.37 m/s, *p* = 0.004) and cadence (98.30 ± 18.89 steps/min vs. 112.50 ± 12.33 steps/min, *p* = 0.019), along with increased step width (13.23 ± 3.92 cm vs. 8.87 ± 2.10 cm, *p* < 0.001) and double support time (38.54 ± 10.26% vs. 22.68 ± 3.27%, *p* < 0.001) relative to controls. Although stride length was shorter in the KOA group (0.97 ± 0.62 m vs. 1.32 ± 0.55 m), this difference was not statistically significant (*p* = 0.084), though a moderate effect size was observed (Cohen's *d* = 0.788). Step length asymmetry was significantly higher in the KOA group (7.67 ± 4.83% vs. 2.75 ± 1.17%, *p* < 0.001).

#### 3.2.2 Comparison of affected KOA side with control group

As detailed in Table 2, significant differences in joint kinematics were observed between the affected side in the KOA group and the control group's bilateral mean. Pelvic kinematics showed differences in tilt (−5.10 ± 5.59° vs. −3.25 ± 5.74°, *p* < 0.001), list (−0.86 ± 3.18° vs. −3.90 ± 4.74°, *p* < 0.001), and rotation (4.00 ± 6.26° vs. 5.39 ± 5.16°, *p* = 0.007). The KOA group displayed reduced peak hip flexion (20.25 ± 7.72°), extension (−11.96 ± 6.49°), adduction (1.63 ± 4.52°), and abduction (−5.17 ± 4.43°), all with *p* < 0.001. Hip internal rotation was significantly increased (−7.65 ± 6.33° vs. −3.02 ± 5.85°, *p* < 0.001), while external rotation was also larger, though not statistically significant (*p* = 0.093). The affected side also exhibited significantly lower peak knee flexion (37.39 ± 13.69° vs. 50.60 ± 8.78°, *p* < 0.001) and extension (0.30 ± 3.21° vs. 2.50 ± 4.90°, *p* < 0.001). At the ankle, the KOA group showed reduced dorsiflexion (6.49 ± 12.35°, *p* < 0.001) and increased plantarflexion (−8.94 ± 9.91°, *p* < 0.001). Subtalar eversion was also reduced (−4.60 ± 11.44° vs. −10.37 ± 10.86°, *p* < 0.001).

**Table 2:** Comparison of peak joint angles between control and experimental groups.

Joint	Peak value (Degree)	Bilateral average of control Mean (SD)	Affected side Mean (SD)	Unffected side Mean (SD)	*p*-Value1	*p*-Value2
Pelvis	Tilt	−3.25 (5.74)	−5.10 (5.59)	−2.12 (5.56)	0.001*	0.622
	List	−3.90 (4.74)	−0.86 (3.18)	−3.01 (3.15)	<0.001*	<0.001*
	Rotation	5.39 (5.16)	4.00 (6.26)	0.40 (7.19)	0.007*	<0.001*
Hip	Flexion	23.07 (7.06)	20.25 (7.72)	17.75 (7.54)	<0.001*	0.008*
	Extension	−14.81 (6.82)	−11.96 (6.49)	−10.92 (8.34)	<0.001*	0.003*
	Adduction	5.32 (4.64)	1.63 (4.52)	3.01 (5.33)	<0.001*	0.887
	Abduction	−7.35 (5.13)	−5.17 (4.43)	−5.31 (11.36)	<0.001*	0.051
	Internal Rotation	−3.02 (5.85)	−7.65 (6.33)	−9.63 (7.69)	<0.001*	<0.001*
	External Rotation	−11.41 (8.15)	−13.47 (6.77)	−20.46 (12.61)	0.093	0.007*
Knee	Flexion	50.60 (8.78)	37.39 (13.69)	36.13 (16.51)	<0.001*	<0.001*
	Extension	2.50 (4.90)	0.30 (3.21)	0.05 (2.75)	<0.001*	<0.001*
Ankle	Dorsiflexion	6.88 (14.13)	6.49 (12.35)	8.68 (12.00)	<0.001*	<0.001*
	Plantarflexion	−5.43 (19.55)	−8.94 (9.91)	−7.82 (10.45)	<0.001*	0.022*
Subtalar	Inversion	2.88 (12.84)	5.37 (12.79)	13.39 (11.06)	0.082	<0.001*
	Eversion	−10.37 (10.86)	−4.60 (11.44)	0.69 (18.75)	<0.001*	0.258

* *p*-value <0.05 is considered statistically significant.

*p*-Value1: between affected side of experiment group and bilateral average of control group.

*p*-Value2: between unaffected side of experiment group and bilateral average of control group.

#### 3.2.3 Comparison of unaffected KOA side with control group

Significant kinematic differences were also observed on the unaffected side of the KOA group. Compared to controls, pelvic list (−3.01 ± 3.15° vs. −3.90 ± 4.74°, *p* < 0.001) and pelvic rotation (0.40 ± 7.19° vs. 5.39 ± 5.16°, *p* < 0.001) were altered. Hip flexion (17.75 ± 7.54°, *p* = 0.008) and extension (−10.92 ± 8.34°, *p* = 0.003) angles were significantly lower. Hip internal (−9.63 ± 7.69°, *p* < 0.001) and external rotation (−20.46 ± 12.61°, *p* < 0.001) were both significantly greater than in controls. Knee flexion (36.13 ± 16.51°) and extension (0.05 ± 2.75°) were reduced (both *p* < 0.001). Ankle dorsiflexion (8.68 ± 12.00°, *p* < 0.001) and plantarflexion (−7.82 ± 10.45°, *p* = 0.022) were significantly altered. Subtalar inversion was elevated (13.39 ± 11.06°, *p* < 0.001).

### 3.3 Inter-group gait cycle comparison

**Figure 2:**
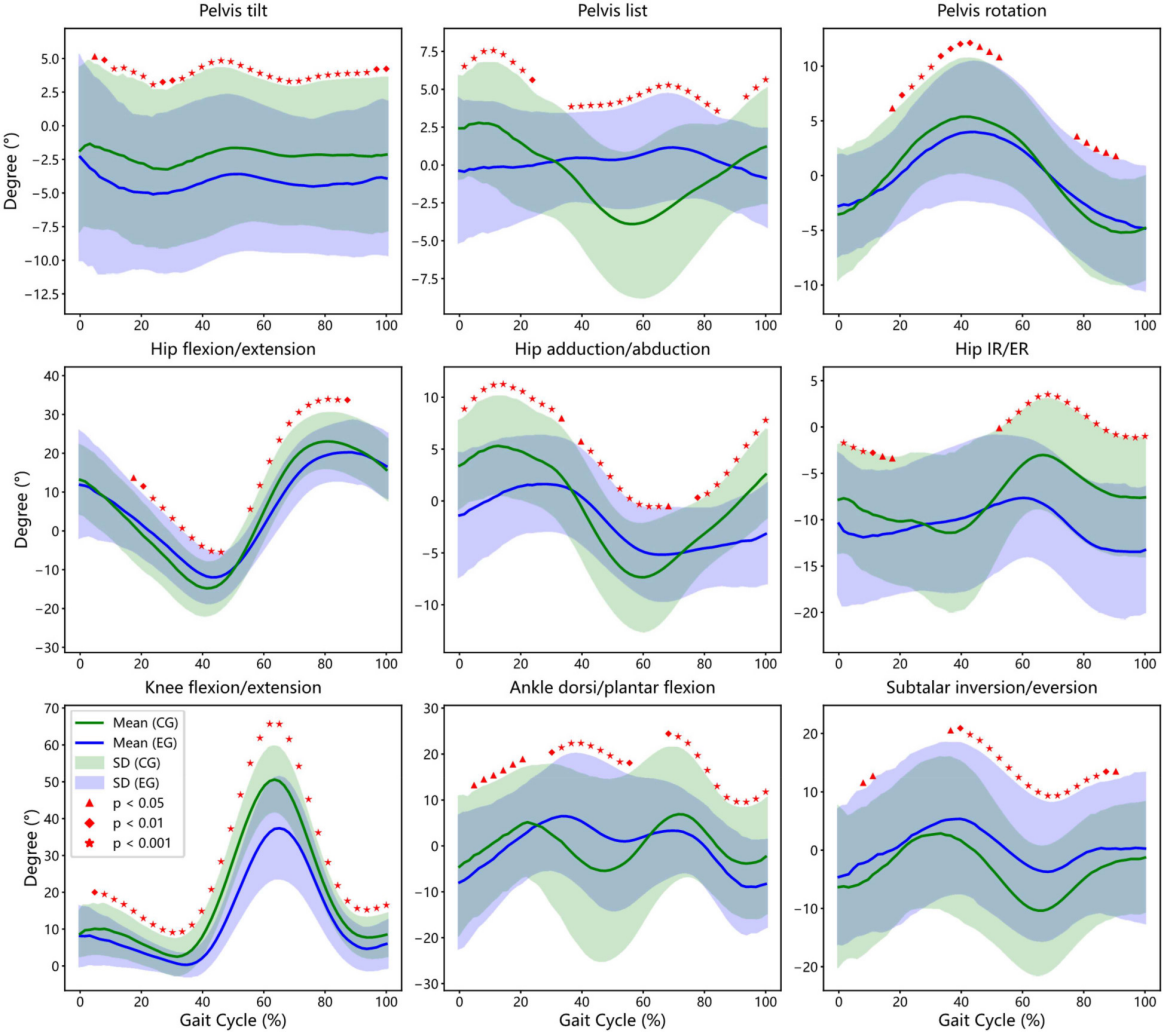
Inter-group gait cycle comparison: affected Side of experimental group (EG) vs. Bilateral Average of Control Group (CG). This figure illustrates the bootstrap confidence intervals and regions of statistically significant differences between patients and controls for key gait parameters, including Pelvis, Hip, Knee, Ankle and Subtalar joint.

A detailed phase-based analysis was performed to compare joint kinematics throughout the entire gait cycle between KOA patients and healthy controls (see Figure 2).

#### 3.3.1 Pelvic kinematic

Compared to controls, KOA patients exhibited a persistent increase in anterior pelvic tilt across nearly the entire gait cycle (3%–100%, *p* < 0.05), indicating compensatory forward trunk positioning potentially related to reduced knee extension. Lateral pelvic tilt (list) showed reduced cyclical modulation in the KOA group, with significant differences observed during early stance and mid-swing phases (0%–24%, 35%–86%, and 92%–100%). Pelvic rotation was significantly reduced during mid-stance and pre-swing phases (17%–49% and 78%–92%), suggesting compromised transverse plane mobility.

#### 3.3.2 Hip kinematics

KOA participants demonstrated a biphasic deviation in sagittal-plane hip kinematics. Specifically, greater hip flexion was noted during early stance (17%–48%, *p* < 0.05), followed by a significant reduction during terminal stance and initial swing (64%–87%). Frontal-plane hip motion showed attenuated adduction–abduction excursions across the gait cycle, while transverse-plane internal and external rotation amplitudes were consistently reduced, with statistical differences spanning multiple gait phases.

#### 3.3.3 Knee kinematic

Although the general flexion-extension profile of the knee joint was preserved in both groups, the KOA group exhibited a marked reduction in flexion range across the majority of the gait cycle (5%–100%, *p* < 0.05), with the most pronounced differences occurring at peak flexion during mid-stance (*p* < 0.001). These observations reflect joint stiffness and limited extensor control during weight-bearing.

#### 3.3.4 Ankle and subtalar kinematic

Significant deviations in ankle kinematics were observed during loading response, mid-stance, and terminal swing (5%–22%, 30%–57%, and 67%–100%), with KOA patients exhibiting altered dorsiflexion profiles. Subtalar joint motion was also impaired, with reduced eversion–inversion excursions during early stance and late swing (6%–13% and 35%–90%, *p* < 0.05), suggesting compromised frontal-plane shock absorption. These findings illustrate that KOA-related joint dysfunction extends beyond the knee, affecting the timing and amplitude of pelvic, hip, and ankle motion—indicative of systemic gait adaptations driven by mechanical and neuromuscular factors.

### 3.4 Intra-group gait comparison (affected vs. unaffected side in KOA group)

**Figure 3:**
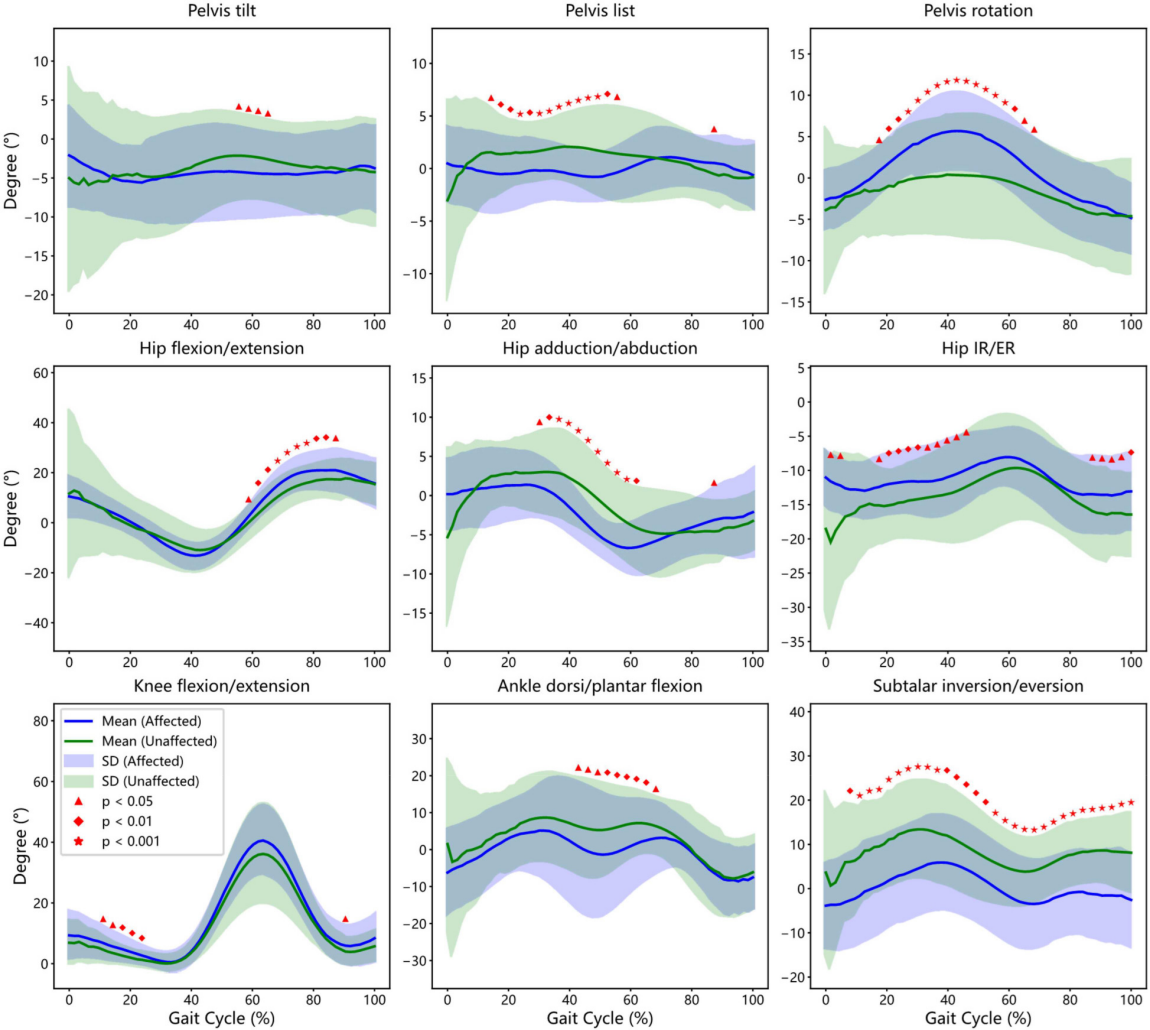
Comparison of gait cycles within the group: affected side in the experimental group (EG) vs. unaffected side in the Experimental Group (CG). This figure illustrates the bootstrap confidence intervals and regions of statistically significant differences between affected side and unaffected side of patients for key gait parameters, including Pelvis, Hip, Knee, Ankle and Subtalar joint.

This analysis compared joint kinematics between the affected and unaffected limbs within KOA patients (see Figure 3).

#### 3.4.1 Pelvic kinematics

While anterior-posterior pelvic tilt profiles were generally similar between limbs, a statistically significant increase in tilt was noted on the affected side during mid-stance to terminal stance (54%–65% of the gait cycle, *p* < 0.05). Lateral pelvic list showed asymmetrical modulation, with the affected side exhibiting a diminished range during 14%–57% of the cycle (*p* < 0.05). Additionally, pelvic rotation was greater on the affected side, with significant inter-limb differences spanning 17%–68% of the gait cycle, indicating potential compensatory trunk movement to mitigate knee instability.

#### 3.4.2 Hip kinematics

Despite grossly similar flexion-extension trajectories between sides, significant differences were detected in specific phases. Affected limb hip flexion-extension angles deviated from the unaffected side during 59%–89% of the cycle (*p* < 0.05), coinciding with the swing-to-stance transition. Reduced adduction and internal rotation were observed on the affected side during 30%–63% and 16%–46%, respectively, while elevated external rotation appeared during terminal swing (87%–100%), suggesting altered femoral control during swing and loading phases.

#### 3.4.3 Knee kinematics

Although both limbs followed a comparable flexion-extension profile, knee flexion angles were significantly reduced on the affected side during early stance (11%–25% of the gait cycle, *p* < 0.05). This reduced dynamic range may reflect a protective strategy to minimize load and pain during weight acceptance (see Table 2), corroborating previous reports of asymmetric extensor activity and joint stiffening (20, 21).

#### 3.4.4 Ankle and subtalar kinematics

Ankle kinematics showed mild temporal differences, with the affected limb exhibiting altered dorsiflexion-plantarflexion transitions during mid-stance to pre-swing (41%–68%, *p* < 0.05). More strikingly, subtalar joint motion revealed consistent and significant reductions in varus–valgus range on the affected side throughout most of the gait cycle (6%–100%, *p* < 0.05), particularly during early stance (6%–13%) and terminal stance to swing (35%–90%). These differences may reflect compromised rearfoot stability and proprioceptive control, which has been shown to affect balance and compensatory adjustments in KOA populations (22). Together, these intra-limb differences highlight the presence of functionally significant compensatory adaptations in KOA, even in the absence of overt asymmetry on visual inspection.

## 4 Discussion

### 4.1 Feasibility, validation, and clinical applicability of OpenCap

This study evaluated the feasibility and clinical applicability of OpenCap, a smartphone-based motion capture system, for assessing gait alterations in patients with knee osteoarthritis (KOA). OpenCap enabled efficient data collection, with each participant completing a full session—including static posture and dynamic trials—within approximately 6–8 min. Its operational simplicity, combined with affordability and satisfactory accuracy, presents significant advantages over traditional marker-based systems, particularly in clinical environments with limited resources. Our data processing workflow retained the complete gait cycle through automated point selection, segmentation, noise reduction, and smoothing, supporting the feasibility of routine gait analysis in hospital settings.

OpenCap offers practical advantages over other markerless systems. Compared to VNect, which relies on manual calibration of camera parameters and human body proportions (taking approximately 30 min), OpenCap employs an automatic calibration algorithm that reduces setup time to about 5 min. For dynamic motion capture tasks, OpenCap has demonstrated particularly strong performance: the measurement error for knee flexion angle was only 3.2°, significantly outperforming VIBE, which reported 7.8° error (15). These features make OpenCap especially attractive for time-efficient, labor-saving, and scalable gait analysis.

Prior validation studies further underscore OpenCap's reliability. For instance, the Stanford University team compared OpenCap using two iPhones to marker-based motion capture and force plate analysis in ten healthy adults performing various activities, reporting a mean absolute error (MAE) of 4.5°, indicating strong agreement (13, 16). Wang et al. assessed OpenCap's accuracy in KOA patients, finding a grand mean root mean square error (RMSE) of 6.1° and an intraclass correlation coefficient (ICC) of 0.67 (17). While these studies focused primarily on technical performance, they did not explore multi-joint compensatory mechanisms or clinical group differences, which our work addresses.

### 4.2 Gait abnormalities in KOA patients

Consistent with prior meta-analyses (11, 23), our study found KOA patients demonstrated slower gait speed, shorter stride length, increased step width, and prolonged double support time. These features likely reflect compensatory strategies to maintain balance and reduce joint load under conditions of pain or instability. In particular, the broader base of support and altered limb loading patterns suggest attempts to enhance postural control. Kinematic analysis revealed significantly reduced peak flexion and extension angles at the knee throughout the gait cycle. Unlike static clinical assessments, which often evaluate passive joint range, dynamic gait analysis captures the actual reduction in active knee motion due to stiffness and pain during ambulation (21, 24).

### 4.3 Individual variability and disease severity

A notable finding was the substantial inter-individual variability within the KOA group. Some patients exhibited gait parameters close to healthy controls, while others deviated markedly. This variability likely reflects heterogeneity in disease severity (KL grades 2–4), pain levels, and progression stage (9, 25). Such differences underscore the need for personalized rehabilitation approaches that consider individual compensatory patterns rather than a one-size-fits-all model. These diverse adaptations highlight the complexity of KOA pathology and the importance of stratifying patients based on functional gait metrics alongside traditional clinical assessments.

### 4.4 Multi-joint and inter-limb compensatory mechanisms

Compensatory mechanisms in KOA include reduced knee joint mobility, increased pelvic tilt, and exaggerated hip internal rotation, which help maintain balance and reduce pain during locomotion (20, 21, 24). These adaptations often arise from neuromuscular imbalances, joint instability, and altered mechanical loading (26, 27). Identifying and quantifying such compensation strategies is crucial for understanding disease severity and guiding clinical interventions.

Our data indicate that gait alterations extend beyond the knee joint, affecting hip and ankle mechanics as well. Specifically, we observed reduced sagittal and frontal plane mobility, increased transverse plane rotation, and excessive plantarflexion in both affected and unaffected limbs. For example, increased hip internal rotation and pelvic anterior tilt may serve as compensations to preserve foot trajectory and forward progression despite limited knee extension during stance. These proximal adaptations shift load-bearing demands to the hip and trunk, helping maintain walking continuity under joint dysfunction (28, 29). Furthermore, side-to-side comparisons revealed abnormal kinematics in the ostensibly unaffected limb, particularly reduced knee mobility and altered pelvic and hip rotations. This likely reflects a neuro-mechanical rebalancing aimed at maintaining bilateral symmetry and locomotor efficiency, potentially at the cost of reduced range of motion on the unaffected side (30).

Increased pelvic tilt combined with reduced lateral pelvic list suggests weakness in hip abductor muscles, especially the gluteus medius, manifesting as a Trendelenburg-like gait pattern (31, 32). This adaptation increases mechanical load on the contralateral limb and may accelerate degenerative changes over time. Exaggerated hip rotation and excessive plantarflexion, particularly through modulation of Achilles tendon tension, may stabilize the affected knee but risk increasing ankle joint load and subsequent cartilage degeneration (33).

### 4.5 Clinical implications for diagnosis and rehabilitation

Our findings reinforce the value of dynamic gait assessment over static measures in KOA evaluation. The observed compensatory strategies highlight the systemic biomechanical adaptations involved in the disease and the importance of capturing these in real-world movement. OpenCap offers a practical tool for longitudinal monitoring, preoperative evaluation, and postoperative rehabilitation management in KOA. It allows clinicians to identify asymmetries and dysfunctional movement patterns that may persist even after surgical interventions such as knee arthroplasty, thereby informing targeted therapies to improve functional outcomes (34, 35). Moreover, prior work demonstrates that OpenCap can simulate muscle activation with up to 75% accuracy compared to electromyography (14). Integration of kinematic and neuromuscular data in clinical workflows could further elucidate compensatory mechanisms and optimize rehabilitation.

### 4.6 Limitations and future directions

Despite its advantages, this study has limitations. OpenCap's accuracy can be influenced by environmental factors such as occlusion, lighting, and camera placement, with joint localization errors ranging from 2 to 5 mm in low-contrast settings (35, 36). Careful control of these conditions is necessary in clinical use. Our sample size, while sufficient for group comparisons, may not fully represent the spectrum of compensatory strategies across KOA populations. Additionally, we did not stratify gait features by age, gender, pain intensity, or KOA severity—factors that warrant exploration in future studies to better tailor interventions. Future research should also integrate surface electromyography with OpenCap to investigate neuromuscular activation patterns underlying biomechanical adaptations, further advancing personalized treatment strategies.

## 5 Conclusion

This study demonstrates that OpenCap, a smartphone-based motion capture system, provides a feasible, accurate, and cost-effective approach for clinical gait analysis in patients with knee osteoarthritis (KOA). OpenCap enables rapid collection of dynamic gait data, capturing key abnormalities such as reduced gait speed, altered stride characteristics, and multi-joint compensatory patterns involving the pelvis, hip, knee, and ankle.

Importantly, this system reveals biomechanical adaptations not only in the affected limb but also in the contralateral side, highlighting the bilateral and systemic nature of KOA-related gait changes. By offering accessible, dynamic assessment beyond traditional static clinical measures, OpenCap supports early diagnosis, personalized rehabilitation planning, and postoperative monitoring.

While limitations exist regarding environmental sensitivity and sample diversity, OpenCap's scalability and ease of implementation make it a valuable tool for integrating comprehensive gait analysis into routine clinical practice. Future work combining OpenCap with neuromuscular assessments may further refine individualized treatment strategies and improve functional outcomes for KOA patients.

## Data Availability

The original contributions presented in the study are included in the article/Supplementary Material, further inquiries can be directed to the corresponding authors.
